# Analysis of the Virulence Profile and Phenotypic Features of Typical and Atypical Enteroaggregative *Escherichia coli* (EAEC) Isolated From Diarrheal Patients in Brazil

**DOI:** 10.3389/fcimb.2020.00144

**Published:** 2020-04-22

**Authors:** Regiane C. B. Dias, Rodrigo H. S. Tanabe, Melissa A. Vieira, Maria C. Cergole-Novella, Luís F. dos Santos, Tânia A. T. Gomes, Waldir P. Elias, Rodrigo T. Hernandes

**Affiliations:** ^1^Universidade Estadual Paulista (UNESP), Instituto de Biociências, São Paulo, Brazil; ^2^Laboratório Regional de Santo André, Instituto Adolfo Lutz, São Paulo, Brazil; ^3^Centro de Bacteriologia, Instituto Adolfo Lutz, São Paulo, Brazil; ^4^Departamento de Microbiologia, Imunologia e Parasitologia, Universidade Federal de São Paulo- Escola Paulista de Medicina (UNIFESP-EPM), São Paulo, Brazil; ^5^Laboratório de Bacteriologia, Instituto Butantan, São Paulo, Brazil

**Keywords:** EAEC, virulence factors, diarrhea, diagnosis, antimicrobial resistance

## Abstract

Enteroaggregative *Escherichia coli* (EAEC) is an important agent of acute and persistent diarrhea in children and adults worldwide. Here we report a characterization of 220 EAEC isolates, 88.2% (194/220) of which were typical and 11.8% (26/220) were atypical, obtained from diarrheal patients during seven years (2010-2016) of epidemiological surveillance in Brazil. The majority of the isolates were assigned to phylogroups A (44.1%, 97/220) or B1 (21.4%, 47/220). The aggregative adherence (AA) pattern was detected in 92.7% (204/220) of the isolates, with six of them exhibiting AA concomitantly with a chain-like adherence pattern; and *agg5A* and *agg4A* were the most common adhesin-encoding genes, which were equally detected in 14.5% (32/220) of the isolates. Each of 12 virulence factor-encoding genes (*agg4A, agg5A, pic, aap, aaiA, aaiC, aaiG, orf3, aar, air, capU*, and *shf*) were statistically associated with typical EAEC (*P* < 0.05). The genes encoding the newly described aggregate-forming pili (AFP) searched (*afpB, afpD, afpP*, and *afpA2*), and/or its regulator (*afpR*), were exclusively detected in atypical EAEC (57.7%, 15/26), and showed a significant association with this subgroup of EAEC (*P* < 0.001). In conclusion, we presented an extensive characterization of the EAEC circulating in the Brazilian settings and identified the *afp* genes as putative markers for increasing the efficiency of atypical EAEC diagnosis.

## Introduction

Enteroaggregative *Escherichia coli* (EAEC) is one of the six diarrheagenic *E. coli* (DEC) pathotypes, which is defined as *E. coli* isolates that produce the aggregative adherence pattern (AA) on cultured epithelial cells (HeLa and/or HEp-2) *in vitro* (Nataro and Kaper, 1998; Kaper et al., 2004; Croxen et al., 2013). The AA pattern is characterized by a bacterial arrangement that resembles stacked bricks on both epithelial cells and the coverslip (Nataro et al., 1987). EAEC is a cause of diarrheal disease in children and adults, living in both developing and developed countries, and are associated with cases of persistent diarrhea and diarrheal outbreaks worldwide (Huang et al., 2006; Estrada-Garcia and Navarro-Garcia, 2012; Hebbelstrup Jensen et al., 2014), besides the fact that EAEC can also be isolated from asymptomatic subjects (Gioppo et al., 2000; Araujo et al., 2007; Lima et al., 2013, 2019a; Dias et al., 2016; Durand et al., 2016).

Previous studies have shown that EAEC isolates can be identified in several serotypes (Zamboni et al., 2004; Boisen et al., 2012; Lima et al., 2019b), and are distributed across the four main *E. coli* phylogroups (A, B1, B2, and D) (França et al., 2013; Imuta et al., 2016). Moreover, high rates of resistance to ampicillin and trimethoprim-sulphamethoxazole, but not to quinolones, have been observed (Aslani et al., 2011; Ikumapayi et al., 2017).

The pathogenicity of EAEC is characterized by the colonization of the intestinal epithelium, followed by the production and secretion of enterotoxins and cytotoxins, which result in secretory diarrhea (Nataro and Kaper, 1998). The establishment of the AA pattern has been associated with the presence of five distinct aggregative adherence fimbriae (AAF/I-AAF/V) (Nataro et al., 1992; Czeczulin et al., 1997; Bernier et al., 2002; Boisen et al., 2008; Jønsson et al., 2015), which belong to the chaperone-usher subclass, as well as afimbrial adhesins (Monteiro-Neto et al., 2003; Bhargava et al., 2009). Very recently, a type IV pilus, termed aggregate-forming pili (AFP), encoded by genes located in the *afp* operon, was identified to be responsible for the establishment of the AA pattern by a hybrid EAEC/STEC (Shiga-toxin producing *E. coli*) isolate of serotype O23:H8, that lacks the AAFs described so far (Lang et al., 2018).

Once adhered to the intestinal epithelium, EAEC produces several enterotoxins and cytotoxins, which include: the enteroaggregative *E. coli* heat-stable enterotoxin (EAST-1), encoded by the *astA* gene, the plasmid-encoded toxin (Pet), the protein involved in colonization (Pic), and autotransporter proteases such as SigA and SepA, which were initially described in *Shigella flexneri*. Another important virulence factor associated with the pathogenicity of EAEC is the protein dispersin (Aap), which facilitates bacterial dispersion in the intestinal epithelium (Sheikh et al., 2002). Dispersin secretion occurs via an ATP-binding cassette (ABC) transporter system, encoded by a cluster of five genes (*aatPABCD*) located in the aggregative adherence plasmid (pAA). The *aatA* gene, which corresponds to the former EAEC probe CVD432, is one the most used target for EAEC identification nowadays (Baudry et al., 1990; Nishi et al., 2003; Lozer et al., 2013).

The AggR protein, encoded by the *aggR* gene located in the pAA, is a global virulence regulator, which activates the expression of several plasmidial and chromosomal virulence factor-encoding genes in the EAEC 042 prototype strain, such as the AAFs, as well as a chromosomal pathogenicity island (PAI), termed *aaiA*-*aaiP*, which encodes a Type VI Secretion System (T6SS) (Dudley et al., 2006; Morin et al., 2013). Some studies employed genes of the *aaiA*-*aaiP* operon, such as *aaiA, aaiC*, and *aaiG*, as an additional chromosomal marker to enhance the efficiency of EAEC diagnosis (Lima et al., 2013; Andrade et al., 2014; Havt et al., 2017; Hebbelstrup Jensen et al., 2018). Based on the presence of the *aggR* gene, EAEC isolates can be subgrouped into typical (*aggR*^+^) and atypical (*aggR*^−^) (Kaper et al., 2004).

Moreover, other potential virulence factor-encoding genes that may contribute to the pathogenicity of EAEC isolates, such as *aar* (AggR-activated regulator), *shf* (which encodes a protein involved in biofilm formation initially identified in *Shigella flexneri*), *air* (Enteroaggregative immunoglobulin repeat protein), and *capU* (which encodes a homologous hexosyltransferase) have been described (Czeczulin et al., 1999; Sheikh et al., 2006; Santiago et al., 2017).

The goal of this study was to better understand the diversity of typical and atypical EAEC isolates, obtained from diarrheal patients in Brazil, regarding the somatic (O) and flagellar (H) antigens determination (serotypes), phylogenetic classification, presence of 25 virulence factors-encoding genes (e.g., adhesins, toxins, secreted proteases, type VI secretion system), adherence pattern produced on HeLa cells, and the susceptibility to 11 distinct antimicrobial drugs.

## Materials and Methods

### Enteroaggregative *Escherichia coli* (EAEC) Isolates Used in This Study

A total of 220 EAEC isolates, obtained from stool samples of sporadic cases of diarrhea occurred in Brazil, were included in this study. Of note, 203 EAEC isolates were obtained over 6-years of active epidemiological surveillance (2011–2016), performed at the Instituto Adolfo Lutz (IAL), a public health laboratory and the Brazilian reference center for diarrheagenic *E. coli* identification, as previously published (Ori et al., 2019), as well as 17 EAEC isolates, obtained during the year of 2010, at the same Institute.

In this study, EAEC was defined as *E. coli* isolates that possess the *aatA* gene (former CVD432 probe), and typical and atypical EAEC were differentiated based on the presence of the *aggR* gene in the former group (Kaper et al., 2004).

### Serotyping

The somatic (O) and flagellar (H) antigens from 203 EAEC isolates, obtained from 2011 to 2016, were determined in our previous epidemiological surveillance study (Ori et al., 2019). In the 17 remaining EAEC isolates, obtained during the year of 2010, the O and H antigens were determined by standard agglutination tests, with O (O1–O181) and H (H1–H56) antisera produced at Instituto Adolfo Lutz, São Paulo, Brazil (Ewing, 1986).

### Phylogroup classification

The classification of the 220 EAEC isolates studied into distinct phylogroups (A, B1, B2, C, D, E, and F) and *Escherichia* clades were performed using the quadruplex Polymerase Chain Reaction (PCR) method as previously described by Clermont et al. (2013). Subsequently, EAEC isolates classified in the phylogroup B2, with the following genotype: *arpA*^−^, *chuA*^+^, *yjaA*^−^, and TspE4^+^, and all isolates assigned in the phylogroup F were tested in an additional Triplex PCR, using primers and PCR conditions as recently described (Clermont et al., 2019), in order to confirm these isolates as B2 and F or to reclassify them in the phylogroup G.

### Detection of Virulence Factor-Encoding Genes

The presence or absence of the EAEC diagnosis markers *aatA* and *aggR* was confirmed in all 220 isolates, and subsequently, these isolates were tested for the presence of genes encoding for: adhesins (*aggA, aafA, agg3A, agg4A, agg5A, afpB, afpD, afpP, afpA2, afpR*, and *eibG*), toxins (*pic, pet, astA, sigA*, and *sepA*) and other virulence factors (*aap, aaiA, aaiC, aaiG, orf3, aar, air, capU*, and *shf*), by PCR DNA amplification. PCR was performed using GoTaq Green Master Mix (Promega, Madison, WI, USA) with 0.34 μM of each of the primers. All primer sequences and PCR assay conditions used for the detection of virulence genes are described in the references cited in Table S1. PCR products were subjected to agarose gel electrophoresis, prepared with Tris-borate-EDTA (TBE) buffer, and visualized with SYBR Safe DNA Gel Stain (Invitrogen, CA, USA).

### Antimicrobial Susceptibility Test

Antimicrobial susceptibility assays were performed following the recommendations of the Clinical and Laboratory Standards Institute (CLSI, 2018), using the disk diffusion method on Mueller-Hinton agar (OXOID, UK). The antimicrobial drugs tested were: ampicillin (AMP; 10 μg), amoxicillin-clavulanic acid (AMC; 20/10 μg), cefazolin (CFZ; 30 μg), cefuroxime (CFX; 30 μg), cefotaxime (CTX; 30 μg), cefepime (CPM; 30 μg), imipenem (IPM; 10 μg), gentamicin (GEN; 10 μg), tobramycin (TOB; 10 μg), ciprofloxacin (CIP; 5 μg), and trimetoprim-sulfamethoxazole (SUT, 1.25/23.75 μg). The *E. coli* ATCC 25922 was used as quality control, and the commercial disks were obtained from Cefar Diagnóstica Ltda (SP, Brazil). EAEC multidrug-resistant was defined by the detection of isolates resistant to three or more classes of antibiotics tested (Magiorakos et al., 2012; Chattaway et al., 2017).

### Adherence Assays

**Figure 1:**
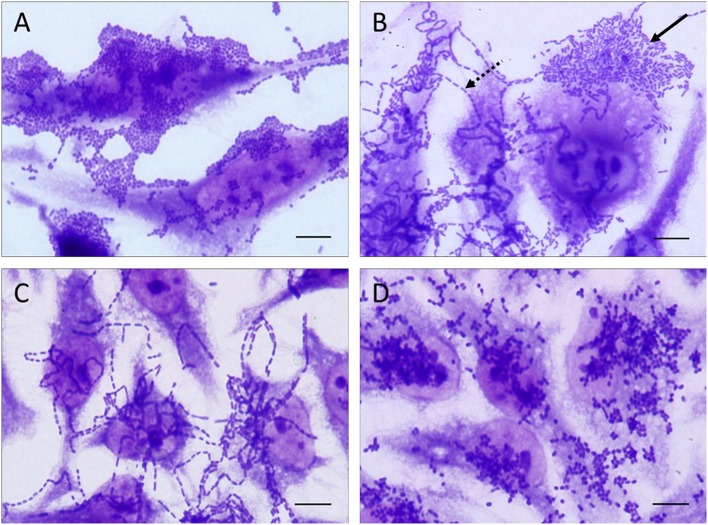
Representative images of the distinct patterns of adherence exhibited by the EAEC isolates studied in adherence assays performed with HeLa cells. The adherence patterns are illustrated as follow: **(A)** aggregative adherence pattern, **(B)** aggregative adherence pattern (solid arrow) occurring concomitantly with the chain-like adherence (dashed arrow), **(C)** chain-like adherence pattern, and **(D)** diffuse adherence pattern. All the adherence patterns illustrated in this picture were observed in adherence assays performed with 3 h of infection. Scale bar = 10 μm.

**Figure 2:**
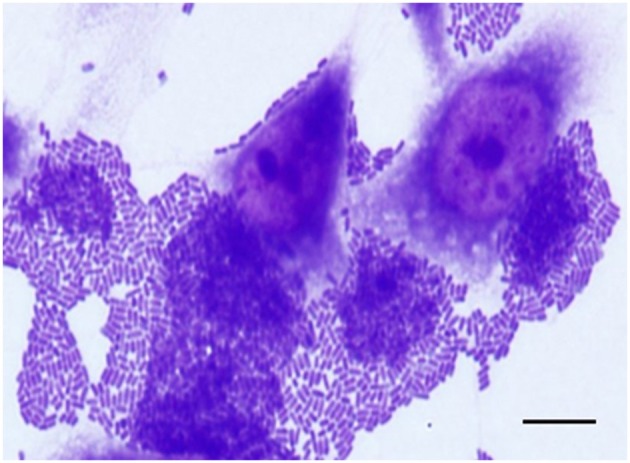
Aggregative adherence (AA) pattern exhibited by an atypical EAEC (*aatA*^+^/*aggR*^−^) isolate (IAL 6006) that harbored the *afp* operon. Note the bacterial stacked brick-like arrangement, characteristic of the AA pattern, on both epithelial cells and coverslip surfaces detected in adherence assay performed with 3 h of infection. Scale bar = 10 μm.

The adherence patterns of the EAEC isolates was evaluated in HeLa cells as previously described (Cravioto et al., 1979), with some modifications. Cells were cultured in DMEM (Dulbecco's Modified Eagle Medium—high glucose; Sigma-Aldrich; St. Louis, MO, USA), supplemented with 10% fetal bovine serum (Gibco; Grand Island, New York, USA), with 1% antibiotic (Penicillin-Streptomycin, Sigma; St. Louis, MO, USA) in 5% CO_2_ at 37°C. For the adherence assay, ~1 × 10^5^ cells were cultivated in a 24-well microplate, using the same medium described above and containing glass coverslips in each well of the microplate, for 48 h until reaching a confluence of ~80%. Then, the cells were washed three times with phosphate-buffered saline (PBS, pH 7.4), 1.0 mL of DMEM, supplemented with 2% fetal bovine serum and 1% D-mannose (Sigma, Saint Louis, MO, USA) was added, the cells were infected with 20 μL of an overnight bacterial suspension in LB broth (containing ~10^8^ CFU/mL), and the adherence assay was performed in 5% CO_2_ at 37°C. After 3 h of incubation, the infected cells were washed six times with PBS and then fixed with methanol (Dinâmica, SP, Brazil) and stained with May-Grünwald (Sigma; St. Louis, MO, USA) and Giemsa (Dinâmica, SP, Brazil) for identification of the adherence pattern by light microscopy analyzes. When the EAEC isolates did not adhere to the epithelial cells or the adherence pattern could not be defined, the adherence assay was repeated in a 6 h bacteria-cell interaction assay, with a washing step after 3 h of incubation, as previously described (Rodrigues et al., 1996), stained with May-Grünwald/Giemsa and then observed by light microscopy. To eliminate potential bias in the adherence pattern classification, each slide was analyzed by three distinct blinded examiners, both to define the adherence pattern exhibited by each EAEC isolate, as well as to select the representative images to be included in Figures 1, 2. In cases of divergence among the three examiners, the EAEC isolate was submitted to a new adherence assay and reevaluated in a blinded-manner until a consensus was obtained.

### Statistical Analyses

Differences observed between typical and atypical EAEC were investigated using a Chi-square with Yates' correction or two-tailed Fisher's exact test, as appropriate, and a significance level of 5% (*P* < 0.05) was adopted to consider the differences statistically significant.

## Results

The majority of the diarrheal patients, from which the EAEC isolates were obtained, were children up to five-years-old (62.7%, 138/220) and from the São Paulo State (66.8%, 147/220), besides EAEC isolates that were obtained from other Brazilian States, such as Minas Gerais (18.6%, 41/220) and Santa Catarina (14.5%, 32/220) (Table S2). We detected 194 typical (88.2%, 194/220) and 26 (11.8%, 26/220) atypical EAEC (Table 1), and observed that 56.8% (125/220) were classified in 39 distinct serogroups, besides the occurrence of O non-typeable (19.5%, 43/220) and rough (23.6%, 52/220) isolates (Table S3). Regarding the serotypes, O15:H2 (4.5%; 10/220), O175:H28 (4.5%; 10/220), O73:H1 (4.1%; 9/220), O153:H2 (3.6%; 8/220), and O21:H2 (3.2%; 7/220) were the most frequent ones, and exclusively detected in the typical EAEC subgroup (Table S3). The classification of the EAEC isolates in the distinct *E. coli* phylogroups, demonstrated that EAEC were assigned mainly in the phylogroups A (44.1%, 97/220), B1 (21.4%, 47/220), D (13.2%, 29/220), and E (10.0%, 22/220) (Table 2), and the number of atypical EAEC identified in the phylogroup A was significantly higher than typical (65.4 vs. 41.2%), with this difference being statistically significant (*P* < 0.05). Moreover, 2.3% (5/220) of the EAEC isolates studied, serotypes O73:H1 (3 isolates) and O15:H18 (2 isolates), were identified in the newly described phylogroup G (Table 2).

**Table 1:** Occurrence of virulence factor-encoding genes in typical and atypical EAEC isolates.

**Genes investigated**	**No. (%) of isolates**			***P*-value^c^^,^^d^**
	**Typical EAEC (*n* = 194)**	**Atypical EAEC (*n* = 26)**	**Total (*n* = 220)**	
**Adhesins**				
*aggA*	6 (3.1)	0	6 (2.7)	NS^d^
*aafA*	18 (9.3)	0	18 (8.2)	NS^d^
*agg3A*	22 (11.3)	0	22 (10.0)	NS^d^
*agg4A*^a^	32 (16.5)	0	32 (14.5)	0.032^d^
*agg5A*^a^	32 (16.5)	0	32 (14.5)	0.032^d^
*afpB*^b^	0	14 (53.8)	14 (6.4)	<0.0001^d^
*afpD*^b^	0	14 (53.8)	14 (6.4)	<0.0001^d^
*afpP*^b^	0	14 (53.8)	14 (6.4)	<0.0001^d^
*afpA2*^b^	0	10 (38.5)	10 (4.5)	<0.0001^d^
*afpR*^b^	0	14 (53.8)	14 (6.4)	<0.0001^d^
*eibG*	1 (0.5)	0	1 (0.5)	NS^d^
**Toxins**				
*pic*^a^	123 (63.4)	4 (15.4)	127 (57.7)	<0.0001^c^
*pet*	20 (10.3)	0	20 (9.1)	NS^d^
*astA*	76 (39.2)	13 (50.0)	89 (40.5)	NS^c^
*sigA*	18 (9.3)	0	18 (8.2)	NS^d^
*sepA*	34 (17.5)	4 (15.4)	38 (17.3)	NS^d^
**Other**				
*aap*^a^	192 (99.0)	18 (69.2)	210 (95.5)	<0.0001^d^
*aaiA*^a^	141 (72.7)	12 (46.2)	153 (69.5)	0.011^c^
*aaiC*^a^	121 (62.4)	3 (11.5)	124 (56.4)	<0.0001^c^
*aaiG*^a^	133 (68.6)	5 (19.2)	138 (62.7)	<0.0001^c^
*orf3*^a^	192 (99.0)	5 (19.2)	197 (89.5)	<0.0001^d^
*aar*^a^	172 (88.7)	8 (30.8)	180 (81.8)	<0.0001^d^
*air*^a^	59 (30.4)	1 (3.8)	60 (27.3)	0.009^c^
*capU*^a^	93 (47.9)	5 (19.2)	98 (44.5)	0.011^c^
*shf*^a^	88 (45.4)	4 (15.4)	92 (41.8)	0.007^c^

a *Virulence genes significantly more prevalent in typical EAEC than in atypical EAEC (P < 0.05)*.

b *Virulence genes significantly more prevalent in atypical EAEC than in typical EAEC (P < 0.0001)*.

c,d *Differences observed between typical and atypical EAEC were tested by using Chi-square test with Yates correlation (c) or two-tailed Fisher's exact test (d), as appropriate*.

*NS: The difference between typical and atypical EAEC was considered not significant*.

**Table 2:** Classification of the typical and atypical EAEC isolates in the distinct *E. coli* phylogroups.

***E. coli* phylogroup**	**Typical EAEC (*n* = 194)**	**Atypical EAEC (*n* = 26)**	**Total (*n* = 220)**	***P*-value**
A	80 (41.2)^a^	17 (65.4)^a^	97 (44.1)	0.034^b^
B1	42 (21.6)	5 (19.2)	47 (21.4)	NS^b^
B2	7 (3.6)	0	7 (3.2)	NS^c^
C	4 (2.1)	0	4 (1.8)	NS^c^
D	28 (14.4)	1 (3.8)	29 (13.2)	NS^c^
E	22 (11.3)	0	22 (10.0)	NS^c^
G	5 (2.6)	0	5 (2.3)	NS^c^
Unknown	6 (3.1)	3 (11.5)	9 (4.1)	NS^c^

a *Statistically significant difference was observed between the two EAEC groups studied (P < 0.05)*.

b,c *Differences observed between typical and atypical EAEC were tested by using Chi-square test with Yates correlation (b) or two-tailed Fisher's exact test (c), as appropriate*.

*NS: The difference between typical and atypical EAEC was considered not significant*.

We also investigated the occurrence of 25 virulence genes encoding adhesins, toxins, secreted proteases, and T6SS (Table 1). Genes encoding the major subunit of the five distinct AAFs were exclusively detected in typical EAEC, with *agg4A* (16.5%, 32/194) and *agg5A* (16.5%, 32/194) being the most frequent ones (Table 1). Interestingly, genes encoding proteins associated with the biogenesis of the newly described AFP adhesin (*afpB, afpD, afpP*, and *afpA2*) and/or its regulator (*afpR*) were exclusively detected in atypical EAEC (57.7%, 15/26), showing a statistical association with this subgroup (*P* < 0.0001). Typical EAEC harbored all five genes encoding for toxins investigated in this study, such as *pic, pet, astA, sigA*, and *sepA*, while atypical EAEC isolates lacked the *pet* and *sigA* genes. Furthermore, *pic* was significantly more frequent in typical than atypical EAEC (63.4 vs. 15.4%, *P* < 0.0001). Additionally, the genes encoding for dispersin (*aap*), T6SS (*aaiA, aaiC*, and *aaiG*) and five other putative virulence factors (*orf3, aar, air, capU*, and *shf*) were detected in typical and atypical, despite their statistical association with the former group (Table 1). The genes encoding the T6SS investigated here (*aaiA, aaiC*, and *aaiG*) were detected in 69.5% (153/220) of the EAEC isolates studied, with the concomitant occurrence of the three genes being the most common genotype (55.5%, 122/220) detected (Table 3). Besides the aforementioned combination, the *aaiA* gene was observed in association with *aaiG* (7.3%, 16/220), *aaiC* (0.9%, 2/220), or not associated with these two genes (5.9%, 13/220) (Table 3).

**Table 3:** Occurrence of the *aai* operon genes in typical and atypical EAEC isolates.

***aai* operon genes**	***N*****°** **(%) of EAEC isolates**		
	**Typical EAEC (*n* = 194)**	**Atypical EAEC (*n* = 26)**	**Total (*n* = 220)**
*aaiA, aaiC, aaiG*	119 (61.3)	3 (11.5)	122 (55.5)
*aaiA, aaiC*	2 (1.0)	0	2 (0.9)
*aaiA, aaiG*	14 (7.2)	2 (7.7)	16 (7.3)
*aaiA*	6 (3.1)	7 (26.9)	13 (5.9)
None	53 (27.3)	14 (53.8)	67 (30.5)

The AA pattern was detected in 92.7% (204/220) of the isolates (Table 4). In 75.9% (167/220) of them, the AA pattern was detected after 3 h of bacteria-cell interaction (Figure 1A), while in 14.1% (31/220) this phenotype was detected using more prolonged periods of incubation (6 h) (Table 4). Curiously, six EAEC isolates (2.7%, 6/220) produced AA concomitantly with long-chain aggregates of bacteria adhered to both HeLa cell surfaces and coverslip (Figure 1B), which is characteristic of an adherence pattern termed chain-like adhesion (CLA). We also observed that 1.4% (3/220) and 0.9% (2/220) of the EAEC isolates produced CLA (Figure 1C) or the diffuse adherence (DA) patterns (Figure 1D), respectively. EAEC isolates exhibiting an undefined (UND) adherence (1.4%, 3/220) or unable to adhere to HeLa cells (3.6%, 8/220), even using more prolonged periods of bacteria-cell interaction, were observed (Table 4). The majority of the typical EAEC isolates (82.4%, 89/108) that harbored one or more of the adhesin-encoding genes investigated were able to produce the AA pattern within 3 h of assay, and the only EAEC that harbored the *eibG* gene produced CLA (Table 4). Interestingly, two typical EAEC isolates harbored the *agg3A* concomitantly with the *agg5A*, and both the isolates were able to produce the AA pattern (Table 4). Among the 26 atypical EAEC, 76.9% (20/26) produced AA, besides isolates exhibiting UND adherence (7.7%, 2/26), or non-adherent (NA) (15.4%, 4/26) (Table 4). Taken into consideration the 14 *afp*^+^ EAEC isolates, 85.7% (12/14) produced AA, while the other two isolates (14.3%, 2/14) were NA (Table 4). The isolate IAL 5749 that harbored only the regulator-encoding gene (*afpR*), but lacked the genes from the *afp* operon investigated, produced a UND adherence pattern to HeLa cells (Table 4).

**Table 4:** Correlation of adhesin-encoding genes and adherence patterns detected in the typical and atypical EAEC isolates studied.

**Adhesin-encoding genes investigated**	**No. of EAEC isolates**	**No. (%) of EAEC exhibiting distinct adherence patterns on HeLa cells^a^**							
		**AA**		**DA**		**AA/CLA**	**CLA**	**UND**	**NA**
		**3 h**	**6 h**	**3 h**	**6 h**	**3 h**	**3 h**	**6 h**	**6 h**
**Typical EAEC (Total)**	**194**	**154 (79.4)**	**24 (12.4)**	**1 (0.5)**	**1 (0.5)**	**6 (3.1)**	**3 (1.5)**	**1 (0.5)**	**4 (2.1)**
*aagA*	6	6 (100.0)	0	0	0	0	0	0	0
*aafA*	18	18 (100.0)	0	0	0	0	0	0	0
*agg3A*	20	18 (90.0)	1 (5.0)	0	0	0	0	1 (5.0)	0
*agg4A*	31	17 (54.8)	7 (22.6)	0	0	4 (12.9)	2 (6.5)	0	1 (3.2)
*agg5A*	30	29 (96.7)	1 (3.3)	0	0	0	0	0	0
*agg3A*+ *agg5A*	2	1 (50.0)	1 (50.0)	0	0	0	0	0	0
*agg4A*+*eibG*	1	0	0	0	0	0	1 (100.0)	0	0
None	86	65 (75.6)	14 (16.3)	1 (1.2)	1 (1.2)	2 (2.3)	0	0	3 (3.5)
**Atypical EAEC (Total)**	**26**	**13 (50.0)**	**7 (26.9)**	**0**	**0**	**0**	**0**	**2 (7.7)**	**4 (15.4)**
*afp* operon	14	11 (78.6)	1 (7.1)	0	0	0	0	0	2 (14.3)
*afpR*	1^b^	0	0	0	0	0	0	1 (100.0)	0
None	11	2 (18.2)	6 (54.5)	0	0	0	0	1 (9.1)	2 (18.2)
**TOTAL**	**220**	**167 (75.9)**	**31 (14.1)**	**1 (0.5)**	**1 (0.5)**	**6 (2.7)**	**3 (1.4)**	**3 (1.4)**	**8 (3.6)**

a *AA, aggregative adherence pattern; CLA, chain-like adherence pattern; AA/CLA, AA observed concomitant with CLA; DA, diffuse adherence pattern; NA, non-adherent EAEC isolates; UND, EAEC isolates exibithing undefined adherence, characterized by the occurrence of few bacteria randomly attached on the HeLa cells surface*.

b *EAEC isolate (IAL 5749) that harbored the afpR gene, but lacked the genes from the afp operon investigated (Table 5)*.

Considering the novelty of the association of the genes of the *afp* operon and/or its regulator (*afpR*) with atypical EAEC, we compiled the main features of these 15 EAEC isolates in Table 5. Most of them were identified in the phylogroup A (80.0%, 12/15) and produced AA in 3 h assay (73.3%, 11/15) (Figure 2), even though they were heterogeneous regarding the serotype, and virulence profile exhibited (Table 5).

**Table 5:** Phenotypic and molecular features of atypical EAEC harboring the *afp* operon gene.

**Atypical EAEC identification**	**Phylogroup**	**Serotype**	**Adherence pattern**		***afp*** **operon**				***afpR***	**Virulence factor-encoding genes**
			**3 h**	**6 h**	***afpB***	***afpD***	***afpP***	***afpA2***		
IAL 5700	A	OR:H33	AA	-	+	+	+	+	+	*aatA*
IAL 5742	Unknown	OR:H45	AA	-	+	+	+	+	+	*aatA, aap, aaiA*
IAL 5744	A	ONT:H-	NA	NA	+	+	+	+	+	*aatA, astA, aap, aaiA*
IAL 5749	B1	OR:H35	NA	UND	-	-	-	-	+	*aatA, aaiA*
IAL 5826	B1	OR:H35	AA	-	+	+	+	+	+	*aatA, aaiA, aar*
IAL 5823	A	ONT:H32	AA	-	+	+	+	+	-	*aatA, aap, capU, shf*
IAL 5846	A	OR:H33	AA	-	+	+	+	+	+	*aatA, aap, orf3, aar*
IAL 5852	A	O80:H10	AA	-	+	+	+	-	+	*aatA, astA, aap, aaiA, orf3, aar*
IAL 5854	A	O80:H10	AA	-	+	+	+	-	+	*aatA, astA, aap, orf3*
IAL 5892	A	OR:H-	AA	-	+	+	+	+	+	*aatA, astA*, aap*, aaiA, capU*
IAL 5898	A	O55:H25	NA	NA	+	+	+	+	+	*aatA*
IAL 5901	A	OR:H10	AA	-	+	+	+	-	+	*aatA, astA, aap*
IAL 5911	A	O55:H25	AA	-	+	+	+	+	+	*aatA, aap*
IAL 6001	A	OR:H10	UND	AA	+	+	+	-	+	*aatA, astA, aap*
IAL 6006	A	ONT:H32	AA	-	+	+	+	+	+	*aatA, aap, capU, shf*

Among the 11 antimicrobial drugs tested, non-susceptible typical EAEC isolates were observed for ampicillin, amoxicillin/clavulanic acid, cefazolin, gentamicin, tobramycin, ciprofloxacin, and trimethoprim/sulfamethoxazole, with the highest resistance rates observed for ampicillin (52.6%, 102/194), and trimethoprim/sulfamethoxazole (33.5%, 65/194) (Table 6). Non-susceptible atypical EAEC were observed only for ampicillin (19.2%, 5/26) and trimethoprim/sulfamethoxazole (19.2%, 5/26) (Table 6). Multidrug resistance phenotype was observed only in two (1.0%, 2/194) of the typical and in none of the atypical EAEC isolates studied (Table S4).

**Table 6:** Antimicrobial susceptibility of the EAEC isolates studied.

**Antimicrobial drugs tested^a^**	**Typical EAEC (*****n*** **=** **194)^b^**			**Atypical EAEC (*****n*** **=** **26)^b^**		
	**S**	**I**	**R**	**S**	**I**	**R**
AMP	92 (47.4)	0	102 (52.6)	21 (80.8)	0	5 (19.2)
AMC	187 (96.4)	6 (3.1)	1 (0.5)	26 (100.0)	0	0
CFZ	159 (82.0)	28 (14.4)	7 (3.6)	24 (92.3)	2 (7.7)	0
CTX	194 (100.0)	0	0	26 (100.0)	0	0
CPM	194 (100.0)	0	0	26 (100.0)	0	0
CFX	194 (100.0)	0	0	26 (100.0)	0	0
IPM	194 (100.0)	0	0	26 (100.0)	0	0
GEN	187 (96.4)	2 (1.0)	5 (2.6)	26 (100.0)	0	0
TOB	190 (97.9)	3 (1.5)	1 (0.5)	26 (100.0)	0	0
CIP	193 (99.5)	0	1 (0.5)	26 (100.0)	0	0
SUT	129 (66.5)	0	65 (33.5)	21 (80.8)	0	5 (19.2)

a *Ampicillin (AMP, 10 μg), amoxicillin-clavulanic acid (AMC, 20/10μg), cefazolin (CFZ, 30 μg), cefuroxime (CFX, 30 μg), cefotaxime (CTX, 30 μg), cefepime (CPM, 30 μg), imipenem (IPM, 10 μg), gentamicin (GEN, 10 μg), tobramycin (TOB, 10 μg), ciprofloxacin (CIP, 5 μg), trimetoprim-sulfamethoxazole (SUT, 1.25 / 23.75 μg)*.

b *Antibiogram interpretation: S, susceptible; I, intermediate; R, resistant*.

## Discussion

The importance of EAEC as an etiological agent of diarrheal diseases has been reinforced in the last decade (Boisen et al., 2012; Lima et al., 2013; Durand et al., 2016; Chattaway et al., 2017; Ori et al., 2019). However, some questions remain to be addressed, such as the use of the adherence pattern as the gold standard for diagnosis, how to identify truly pathogenic isolates in this heterogeneous group of isolates, and which would be the best molecular markers to improve the diagnosis of EAEC, mainly atypical EAEC.

Several of the virulence factor-encoding genes investigated here were statistically more frequent among typical EAEC, which is not surprising since the majority of these genes were identified, and functionally characterized, in the typical EAEC prototype strain 042 (Harrington et al., 2006; Chaudhuri et al., 2010; Hebbelstrup Jensen et al., 2014). Similar findings were also observed by other Brazilian authors that compared typical and atypical EAEC isolates, especially with the *pic, aap, shf*, and *agg4A* genes being more frequent among the typical EAEC isolates (Andrade et al., 2017; Taborda et al., 2018; Guerrieri et al., 2019).

Case-control studies, performed in distinct geographic regions, have been able to demonstrate the association of some genes encoding virulence factors with diarrheal disease. *pic*, which encodes a protein with hemagglutinin and mucinolytic activity *in vitro* (Henderson et al., 1999), was statistically more frequent among diarrheal Peruvian children than in healthy subjects (Durand et al., 2016), as well as *sepA*, encoding an autotransporter protease originally described in *Shigella* spp., was associated with diarrhea in children from Bamako, Mali (Boisen et al., 2012). A case-control study performed in Northeastern Brazil demonstrated the association of *pet* (Plasmid-encoded toxin) and *aafA* (AAF/II fimbrial subunit) with diarrhea (Lima et al., 2013). Moreover, the occurrence of typical EAEC, harboring concomitantly *aatA* and *aaiC*, was more frequent among malnourished than in nourished children (Havt et al., 2017), thus reinforcing the need for greater attention to asymptomatic infections by this pathogen.

In typical EAEC the establishment of the AA pattern is mediated by five distinct AAFs (Jønsson et al., 2017a), and the occurrence of genes associated with the biogenesis of AAF/I-AAF/IV has already been reported among Brazilian EAEC isolates, with their frequency varying according to the geographic regions studied (Elias et al., 1999; Zamboni et al., 2004; Lima et al., 2013; Guerrieri et al., 2019). However, the occurrence of Brazilian EAEC isolates harboring the *agg5A*, alone or concomitantly with *agg3A*, is described for the first time in this study. A study performed with Danish EAEC isolates identified six harboring the major pilin subunits from both AAF/III (*agg3A*) and AAF/V (*agg5A*), with these genes located in a novel pAA variant (Jønsson et al., 2017b).

Recently, Lang and coworkers described a new fimbria, termed aggregate-forming pili (AFP), in a hybrid EAEC/STEC isolate of serotype O23:H8 (Lang et al., 2018). The genes responsible for the biogenesis of AFP have ~52% of nucleotide identity with the genes responsible for encoding the bundle-forming pili (BFP), a type IV pilus that mediates the localized adherence (LA) phenotype of typical enteropathogenic *E. coli* on epithelial cells surface (Scaletsky et al., 1984; Girón et al., 1991; Donnenberg et al., 1992). The *afp* and *bfp* operons have a similar gene organization (Tobe et al., 1999; Lang et al., 2018), despite the presence of an additional *afpA2* gene, that is not required for AFP biogenesis (Lang et al., 2018). The deletion of the complete *afp* operon, *afpA* and *afpR* genes (encoding an AraC-like regulator), but not *afpA2*, led to the loss of fimbriae production and drastically reduced the ability of the EAEC/STEC isolate in adhering to epithelial cells *in vitro* (Lang et al., 2018).

Using an NCBI nucleotide BLAST analysis with the *afp* operon genes as a query sequence, Lang et al. (2018) identified 17 *afp*-positive *E. coli* isolates, which were also positive for EAEC markers, such as *aatA* and *aap*, but lacking *aggR*, which is a genotype of atypical EAEC. This observation motived us to deeply investigate the occurrence of the *afp* genes in other EAEC isolates, including both typical and atypical. To the best of our knowledge, this is the first study to demonstrate the association of genes from the *afp* operon, as well as, a gene encoding its regulator (*afpR*) with the atypical EAEC subgroup, thus providing a new putative molecular marker to increase the diagnostic efficiency of this pathogen.

Moreover, some of the isolates classified as typical EAEC (*aatA*^+^/*aggR*^+^) produced the CLA or DA patterns on HeLa cells (3 and 2 isolates, respectively), similar to the observed in previous studies that compared adherence pattern and molecular markers of the distinct DEC pathotypes (Gomes et al., 1998; Gioppo et al., 2000; Scaletsky et al., 2002; Spano et al., 2017). A Brazilian study demonstrated that *E. coli* isolates exhibiting CLA on epithelial cells harbored genes commonly used as a marker of the EAEC pathotype, i.e., *aatA* and *aggR*, as well as virulence factor-encoding genes, such as *pet, astA*, and *aggC* (Gioppo et al., 2000). These data demonstrate that the EAEC definition is still ambiguous since *E. coli* isolates producing the AA pattern on HeLa cells could be devoid of EAEC molecular markers of this pathotype, as well as *E. coli* positive for such genes could produce adherence patterns distinct from AA. We believe that further studies using whole-genome sequencing analysis, in combination with phenotypic features of the EAEC isolates and clinical information of the patients could provide greater accuracy to the molecular markers necessary for the EAEC diagnosis, as well as, in the identification of truly pathogenic isolates.

In conclusion, although we have characterized the EAEC isolates studied in terms of phylogroups, serotypes, adherence patterns, and genes that encode virulence factors, the main message of this study is the association of the aggregate-forming pili (AFP)-encoding genes with the atypical EAEC subgroup. Our study showed that the *afp* operon was found only in *E. coli* isolates that carry the *aatA* gene, but are devoid of the genes encoding the EAEC virulence regulator and the aggregative adherence fimbriae (*aatA*^+^/*aggR*^−^/AAFs^−^), thus providing a novel putative marker for increasing the efficiency of atypical EAEC diagnosis.

## Data Availability Statement

All datasets generated for this study are included in the article/Supplementary Material.

## Ethics Statement

This study was analyzed and approved by the Botucatu Medical School Ethical Committee for human experimentation (CAAE 09994419.6.1001.5411). Written and informed consent from the participants was not required as approved by the aforementioned Ethics Committee.

## Author Contributions

RH, LS, and WE conceptualized the study. RD, LS, RH, and WE contributed to the formal analysis. RH and RD were responsible for the funding acquisition. RD, RT, MV, MC-N, LS, TG, WE, and RH carried out the investigation. RD, RT, MV, and MC-N worked on the methodology. RH, RD, and LS helped with the project administration. RH, LS, TG, and WE supervised the study. RH, LS, TG, and WE validated the study. RD and RH wrote the original draft. RD, RT, MV, MC-N, LS, TG, WE, and RH reviewed and edited the manuscript.

## Conflict of Interest

The authors declare that the research was conducted in the absence of any commercial or financial relationships that could be construed as a potential conflict of interest.
