# Supplementary material for: Analysis of the Virulence Profile and Phenotypic Features of Typical and Atypical Enteroaggregative Escherichia coli (EAEC) Isolated From Diarrheal Patients in Brazil
Source: Front Cell Infect Microbiol. 2020 Apr 22;10:144. doi: 10.3389/fcimb.2020.00144 (PMC7188757; doi:10.3389/fcimb.2020.00144)
Supplement: Supplementary file 1 [file Data_Sheet_1.PDF]

Table S1. Primers used for detection of virulence factor-encoding genes in the 220 EAEC isolates studied.

Target gene	Sequence (5´- 3´)	Fragment size (bp)	Reference
**Diagnostic markers**			
aatA	CTGGCGAAAGACTGTATCAT<br>CAATGTATAGAAATCCGCTGTT	630	Schmidt et al., 1995
aggR	GTATACACAAAAGAAGGAAGC<br>ACAGAATCGTCAGCATCAGC	254	Toma et al., 2003
**Adhesins**			
aggA	GCGTTAGAAAGACCTCCAATA<br>GCCGGATCCTTAAAAATTAATTCCGGC	432	Savarino et al., 1994
aafA	ACATGCATGCAAAAAATCAGAATGTTTGTT<br>CGGGATCCATTTGTCACAAGCTCAGC	550	Czeczulin et al., 1997
agg3A	GTATCATTGCGAGTCTGGTATTCAG<br>GGGCTGTTATAGAGTAACTTCCAG	462	Bernier et al. (2002)
agg4A	TCCATTATGTCAGGCTGCAA<br>GGCGTTAACGTCTGATTTCC	411	Boisen et al., 2008
agg5A	CATGTTCATTATCTATTAGTTCGCC<br>TCCACCGTACGTCGTCATTA	215	Jønsson et al., 2015
afpA2	ATCGTCTCCGCTGTTAACGG<br>TTTACAAGCATTGGCAGCACCAGC	381	Lang et al., 2018
afpB	TACAACGATGTCAGGGGTGC<br>TTACCCTGTCGCTTTCCACC	420	Lang et al., 2018
afpD	AGGAAGAACGAGGGAGGGAA<br>CCCAGTGGTCAACAGCTTCT	513	Lang et al., 2018
afpP	ATATGGTCGCCACCTTCGAG<br>TCAATGAGGGGCCCATAGGA	586	Lang et al., 2018
afpR	GTGAAGAACATTATTGAAGGGGGC<br>CATCACTTAATCGCCAGCGTT	307	Lang et al., 2018
eibG	ATCGGCTTTCATCGCATCAGGAC<br>CCACAAGGCGGGTATTCGTATC	508	Lu et al., 2006
**Toxins**			
pic	GGGTATTGTCCGTTCCGAT<br>ACAACGATACCGTCTCCCG	1175	Czeczulin et al., 1999
pet	GTGTTTCAACCAGGTTCAACA<br>CCTTCACCAATTTTATGCAGT	1037	Gioppo et al., 2000
sigA	CCGACTTCTCACTTTCTCCCG<br>CCATCCAGCTGCATAGTGTTTG	430	Boisen et al., 2009
sepA	GCAGTGGAAATATGATGCGGC<br>TTGTTCAGATCGGAGAAGAACG	794	Restieri et al., 2007

Table S1. *Continued*

Target gene	Sequence (5´- 3´)	Fragment size (bp)	Reference
**Type VI Secretion System**			
aaiA	CCCACGAGTACCAGATAACG<br>GTTTTCAGGATTGCCATTAG	476	Dudley et al., 2006
aaiC	ATTGTCCTCAGGCATTTCACACG<br>GACACCCCTGATAAACAAC	215	Lima et al., 2013[a]
aaiG	GGGAGTGTTTCAGTCTGGAC<br>TTATCGGTGATAAGACTACCACTT	808	Andrade et al., 2014[b]
**Other genes**			
aap	CTTTTCTGGCATCTTGGGT<br>GTAACAACCCCTTTGGAAGT	232	Czeczulin et al., 1999
shf	ACTTTCTCCCGAGACATTC<br>CTTTAGCGGGAGCATTCAT	613	Czeczulin et al., 1999
orf3	CAGCAACCATCGCATTTCTA<br>CGCATCTTTCAATACCTCCA	121	Boisen et al., 2012
aar	AGCTCTGGAAACTGGCCTCT<br>AACCGTCCTGATTTCTGCTT	108	Boisen et al., 2012
air	TTATCCTGGTCTGTCTCAAT<br>GGTTAAATCGCTGGTTTCTT	600	Havt et al., 2017
capU	CAGGCTGTTGCTCAAATGAA<br>GTTCGACATCCTTCCTGCTC	395	Boisen et al., 2012

[a]The nucleotide bases underlined in the *aaiC* reverse primer represent modifications added from the reference cited.
[b]The reverse primer was designed in this study.

REFERENCES

Andrade, F. B., Gomes, T. A., and Elias, W. P. (2014). A sensitive and specific molecular tool for detection of both typical and atypical enteroaggregative *Escherichia coli*. *J. Microbiol. Methods.* 106:16-18. doi: 10.1016/j.mimet.2014.07.030

Bernier, C., Gounon, P., and Le Bouguénec, C. (2002). Identification of an aggregative adhesion fimbria (AAF) type III-encoding operon in enteroaggregative *Escherichia coli* as a sensitive probe for detecting the AAF-encoding operon family. *Infect Immun.* 70:4302-4311. doi: 10.1128/iai.70.8.4302-4311.2002

Boisen, N., Ruiz-Perez, F., Scheutz, F., Krogfelt, K. A., P. Nataro, J. P. (2009). Short Report: High Prevalence of Serine Protease Autotransporter Cytotoxins among Strains of Enteroaggregative *Escherichia coli*. *Am. J. Trop. Med. Hyg*. 80:294–301.

Boisen, N., Scheutz, F., Rasko, D. A., Redman, J. C., Persson, S., Simon, J., et al. (2012). Genomic characterization of enteroaggregative *Escherichia coli* from children in Mali. *J. Infect. Dis.* 205:431-444. doi: 10.1093/infdis/jir757

Boisen, N., Struve, C., Scheutz, F., Krogfelt, K. A., Nataro, J. P. (2008) New adhesin of enteroaggregative *Escherichia coli* related to the Afa/Dr/AAF family. *Infect. Immun.* 76:3281- 3292.doi: 10.1128/IAI.01646-07.

Czeczulin, J. R., Balepur, S., Hicks, S., Phillips, A., Hall, R., Kothary, M. H., et al. (1997). Aggregative adherence fímbria II, a second fimbrial antigen mediating aggregative adherence in enteroaggregative *Escherichia coli*. *Infect. Immun*. 65:4135-4145.

Czeczulin, J. R., Whittam, T. S., Henderson, I. R., Navarro-Garcia, F., Nataro, J. P. (1999). Phylogenetic analysis of enteroaggregative and diffusely adherent *Escherichia coli*. *Infect. Immun.* 67:2692-2699.

Dudley, E. G., Thomson, N. R., Parkhill, J., Morin, N. P., Nataro, J. P. (2006). Proteomic and microarray characterization of the AggR regulon identifies a pheU pathogenicity island in enteroaggregative *Escherichia coli.Mol. Microbiol*. 61:1267–1282. doi: 10.1111/j.1365-2958.2006.05281.x

Gioppo, N. M., Elias, W. P. Jr., Vidotto, M. C., Linhares, R. E.,Saridakis, H. O., Gomes, T. A., et al. (2000). Prevalence of HEp-2 cell-adherent *Escherichia coli* and characterisation of enteroaggregative *E. coli* and chain-like adherent *E. coli* isolated from children with and without diarrhoea, in Londrina, Brazil. *FEMS Microbiol. Lett*. 190:293-298. doi: 10.1111/j.1574-6968.2000.tb09301.x

Havt, A., Lima, I. F., Medeiros, P. H., Clementino, M. A., Santos, A. K., Amaral, M. S., et al. (2017). Prevalence and virulence gene profiling of enteroaggregative *Escherichia coli* in malnourished and nourished Brazilian children. *Diagn. Microbiol. Infect. Dis*. 89:98-105. doi: 10.1016/j.diagmicrobio.2017.06.024

Jønsson, R., Struve, C., Boisen, N., Mateiu, R. M., Santiago, A. E., Jenssen, H., et al. (2015). Novel Aggregative Adherence Fimbria Variant of Enteroaggregative *Escherichia coli*. *Infect. Immun*. 83: 1396–1405. doi: 10.1128/IAI.02820-14.

Lang, C., Fruth, A., Holland, G., Laue, M., Mühlen, S., Dersch, P., et al. (2018). Novel type of pilus associated with a Shiga-toxigenic *E. coli* hybrid pathovar conveys aggregative adherence and bacterial virulence. *Emerg. Microbes. Infect*. 7:203. doi: 10.1038/s41426-018-0209-8

Lima, I. F., Boisen, N., Quetz Jda, S., Havt, A., de Carvalho, E. B., Soares, A. M., et al. (2013). Prevalence of enteroaggregative *Escherichia coli* and its virulence-related genes in a case-control study among children from north-eastern Brazil. *J. Med. Microbiol.* 62:683-693. doi: 10.1099/jmm.0.054262-0

Lu, Y., Iyoda, S., Satou, H., Satou, H., Itoh, K., Saitoh, T., et al. (2006). A new immunoglobulin-binding protein, EibG, is responsible for the chain-like adhesion phenotype of locus of enterocyte effacement-negative, shiga toxin-producing *Escherichia coli*. *Infect. Immun*. 74:5747-55. doi: 10.1128/IAI.00724-06

Restieri, C., Garriss, G., Locas, M. C., Dozois, C. M. (2007). Autotransporter-Encoding Sequences Are Phylogenetically Distributed among *Escherichia coli* Clinical Isolates and Reference Strains. *Appl. Environ. Microbiol*. 2007 Mar; 73(5): 1553–1562. doi: 10.1128/AEM.01542-06

Savarino, S. J., Fox, P., Deng, Y., Nataro, J. P. (1994). Identification and characterization of a gene cluster mediating enteroaggregative *Escherichia coli* aggregative adherence fimbria I biogenesis. *J. Bacteriol*. 176:4949-57. doi: 10.1128/jb.176.16.4949-4957.1994

Schmidt, H., Knop, C., Franke, S., Aleksic, S., Heesemann, J., Karch, H. (1995). Development of PCR for screening of enteroaggregative *Escherichia coli*. *J. Clin. Microbiol*. 33:701-5.

Toma, C., Lu, Y., Higa, N., Nakasone, N., Chinen, I., Baschkier, A., et al. (2003). Multiplex PCR assay for identification of human diarrheagenic *Escherichia coli*. *J. Clin. Microbiol*. 41:2669-71. doi: 10.1128/jcm.41.6.2669-2671.2003
