# Supplementary material for: Analysis of the Virulence Profile and Phenotypic Features of Typical and Atypical Enteroaggregative Escherichia coli (EAEC) Isolated From Diarrheal Patients in Brazil
Source: Front Cell Infect Microbiol. 2020 Apr 22;10:144. doi: 10.3389/fcimb.2020.00144 (PMC7188757; doi:10.3389/fcimb.2020.00144)
Supplement: Supplementary file 2 [file Data_Sheet_2.PDF]

Table S2. Patient's features and Demographic information regarding the origin of the EAEC isolates studied.

Demographic and Patients features	Typical EAEC (*n* = 194)	Atypical EAEC (*n* = 26)	Total (*n* = 220)
**Age (years)**			
0-5	121 (62.4)	17 (65.4)	138 (62.7)
6-10	2 (1.0)	0	2 (0.9)
11-59	60 (30.9)	8 (30.8)	68 (30.9)
>60	8 (4.1)	1 (3.8)	9 (4.1)
No information	3 (1.5)	0	3 (1.4)
**Sex**			
Female	109 (56.2)	12 (46.2)	121 (55.0)
Male	85 (43.8)	14 (53.8)	99 (45.0)
**Brazilian State of Origin**			
São Paulo (SP)	128 (66.0)	19 (73.1)	147 (66.8)
Minas Gerais (MG)	38 (19.6)	3 (11.5)	41 (18.6)
Santa Catarina (SC)	28 (14.4)	4 (15.4)	32 (14.5)
**Year of isolation**			
2010	14 (7.2)	3 (11.5)	17 (7.7)
2011	7 (3.6)	1 (3.8)	8 (3.6)
2012	23 (11.9)	0	23 (10.5)
2013	29 (14.9)	4 (15.4)	33 (15.0)
2014	38 (19.6)	2 (7.7)	40 (18.2)
2015	30 (15.5)	5 (19.2)	35 (15.9)
2016	53 (27.3)	11 (42.3)	64 (29.1)
