# Supplementary material for: Analysis of the Virulence Profile and Phenotypic Features of Typical and Atypical Enteroaggregative Escherichia coli (EAEC) Isolated From Diarrheal Patients in Brazil
Source: Front Cell Infect Microbiol. 2020 Apr 22;10:144. doi: 10.3389/fcimb.2020.00144 (PMC7188757; doi:10.3389/fcimb.2020.00144)
Supplement: Supplementary file 3 [file Data_Sheet_3.PDF]

Table S3. Serotypes of the typical and atypical EAEC isolates studied.

Serogroup[a]	H type[b]	Typical EAEC (*n* = 194)	Atypical EAEC (*n* = 26)	Total (*n* = 220)
O3	H2	3 (1.5)	0	3 (1.4)
O5	H10	2 (1.0)	0	2 (0.9)
O9	H10	2 (1.0)	0	2 (0.9)
O11	H18	1 (0.5)	0	1 (0.5)
O15	H2	10 (5.2)	0	10 (4.5)
	H18	4 (2.1)	0	4 (1.8)
O21	H2	7 (3.6)	0	7 (3.2)
O25	H4	1 (0.5)	0	1 (0.5)
O38	H25	3 (1.5)	0	3 (1.4)
O43	H2	0	1 (3.8)	1 (0.5)
O44	H18	2 (1.0)	0	2 (0.9)
O45	H45	1 (0.5)	0	1 (0.5)
O55	H21	1 (0.5)	0	1 (0.5)
	H25	0	2 (7.7)	2 (0.9)
O56	H2	1 (0.5)	0	1 (0.5)
O59	H19	2 (1.0)	0	2 (0.9)
	HNM	3 (1.5)	0	3 (1.4)
O65	H31	1 (0.5)	0	1 (0.5)
O68	H1	1 (0.5)	0	1 (0.5)
O73	H1	9 (4.6)	0	9 (4.1)
	H18	4 (2.1)	0	4 (1.8)
	H33	1 (0.5)	0	1 (0.5)
O77	H18	1 (0.5)	0	1 (0.5)
O78	H10	0	2 (7.7)	2 (0.9)
O80	H10	0	3 (11.5)	3 (1.4)
O81	H27	0	1 (3.8)	1 (0.5)
O82	H10	1 (0.5)	0	1 (0.5)
O84	HNM	1 (0.5)	0	1 (0.5)
O86	H2	3 (1.5)	0	3 (1.4)
O99	H6	3 (1.5)	0	3 (1.4)
O104	H4	2 (1.0)	1 (3.8)	3 (1.4)
O106	H18	2 (1.0)	0	2 (0.9)
	HNM	1 (0.5)	0	1 (0.5)
O114	H10	1 (0.5)	0	1 (0.5)
O131	H25	1 (0.5)	0	1 (0.5)
O138	H48	1 (0.5)	0	1 (0.5)
O139	H19	0	1 (3.8)	1 (0.5)

Table S3. *Continued*

Serogroup[a]	H type[b]	Typical EAEC (*n* = 194)	Atypical EAEC (*n* = 26)	Total (*n* = 220)
O153	H2	8 (4.1)	0	8 (3.6)
	HNM	2 (1.0)	0	2 (0.9)
O155	H19	2 (1.0)	0	2 (0.9)
O165	H4	1 (0.5)	0	1 (0.5)
O168	HNM	4 (2.1)	0	4 (1.8)
O175	H18	1 (0.5)	0	1 (0.5)
	H23	1 (0.5)	0	1 (0.5)
	H28	10 (5.2)	0	10 (4.5)
	HND	1 (0.5)	0	1 (0.5)
O176	H33	4 (2.1)	0	4 (1.8)
	H34	1 (0.5)	0	1 (0.5)
O179	HNM	1 (0.5)	0	1 (0.5)
O181	H28	2 (1.0)	0	2 (0.9)
ONT	H1	1 (0.5)	0	1 (0.5)
	H2	2 (1.0)	0	2 (0.9)
	H4	4 (2.1)	0	4 (1.8)
	H10	14 (7.2)	0	14 (6.4)
	H18	3 (1.5)	0	3 (1.4)
	H19	1 (0.5)	0	1 (0.5)
	H21	3 (1.5)	0	3 (1.4)
	H32	0	2 (7.7)	2 (0.9)
	H33	0	1 (3.8)	1 (0.5)
	HND	2 (1.0)	0	2 (0.9)
	HNM	9 (4.6)	1 (3.8)	10 (4.5)
OR	H2	11 (5.7)	0	11 (5)
	H4	2 (1.0)	0	2 (0.9)
	H10	2 (1.0)	2 (7.7)	4 (1.8)
	H18	6 (3.1)	0	6 (2.7)
	H21	0	1 (3.8)	1 (0.5)
	H25	2 (1.0)	0	2 (0.9)
	H33	1 (0.5)	3 (11.5)	4 (1.8)
	H35	0	2 (7.7)	2 (0.9)
	H45	0	1 (3.8)	1 (0.5)
	HND	1 (0.5)	0	1 (0.5)
	HNM	16 (8.2)	2 (7.7)	18 (8.2)

[a]ONT: non-typeable isolates for somatic antigen O with tested antisera (O1 to O181), OR: rough.
[b]HNM: isolates non-motile, HND: non-typeable flagellar antigen H with tested antisera (H1 to H56).
