# Supplementary material for: Analysis of the Virulence Profile and Phenotypic Features of Typical and Atypical Enteroaggregative Escherichia coli (EAEC) Isolated From Diarrheal Patients in Brazil
Source: Front Cell Infect Microbiol. 2020 Apr 22;10:144. doi: 10.3389/fcimb.2020.00144 (PMC7188757; doi:10.3389/fcimb.2020.00144)
Supplement: Supplementary file 4 [file Data_Sheet_4.PDF]

Table S4. Combinations of antimicrobial resistance observed in the EAEC isolates investigated in this study.

**Classes of antimicrobial drugs:**				**Typical EAEC**	**Atypical EAEC**
**β-lactam**	**Aminoglycoside**	**Quinolone**	**Folate pathway inhibitors**	**(*n* = 194)**	**(*n* = 26)**
AMP	-	-	-	39 (20.1)	2 (7.7)
AMP, AMC, CFZ	-	-	SUT	1 (0.5)	0
AMP, CFZ	-	-	-	1 (0.5)	0
AMP, CFZ	-	-	SUT	5 (2.6)	0
AMP	GEN	-	-	3 (1.5)	0
AMP	GEN	CIP	SUT	1 (0.5)[a]	0
AMP	GEN, TOB	-	SUT	1 (0.5)[a]	0
AMP	-	-	SUT	51 (26.3)	3 (11.5)
-	-	-	SUT	6 (3.1)	2 (7.7)

[a]Multidrug resistance (MDR) EAEC was defined as EAEC isolates showing resistance to three or more distinct classes of antimicrobial drugs.
